# Cylindrical Piezoelectric PZT Transducers for Sensing and Actuation

**DOI:** 10.3390/s23063042

**Published:** 2023-03-11

**Authors:** Ata Meshkinzar, Ahmed M. Al-Jumaily

**Affiliations:** Institute of Biomedical Technologies, Auckland University of Technology, 6-24 St Paul St, Auckland 1010, New Zealand

**Keywords:** sensing, actuation, piezoelectric PZT transducers, cylindrical transducers, stepped-thickness transducers, biomedical transducers, food industry transducers

## Abstract

Piezoelectric transducers have numerous applications in a wide range of sensing and actuation applications. Such a variety has resulted in continuous research into the design and development of these transducers, including but not limited to their geometry, material and configuration. Among these, cylindrical-shaped piezoelectric PZT transducers with superior features are suitable for various sensor or actuator applications. However, despite their strong potential, they have not been thoroughly investigated and fully established. The aim of this paper is to shed light on various cylindrical piezoelectric PZT transducers, their applications and design configurations. Based on the latest literature, different design configurations such as stepped-thickness cylindrical transducers and their potential application areas will be elaborated on to propose future research trends for introducing new configurations that meet the requirements for biomedical applications, the food industry, as well as other industrial fields.

## 1. Introduction

Piezoelectric transducers have applications as sensors and actuators in numerous fields, including the biomedical, food industry and nondestructive testing (NDT), among many other industrial applications [1]. Depending on the application, requirements and material properties, a variety of geometries and configurations have been used, including but not limited to rectangular or circular plates, arrays of transducers and cylinders from micro- to macroscale [2,3,4,5,6,7,8,9,10,11,12,13,14,15,16,17,18]. In spite of the fact that it has been endeavored to employ the most suitable material for each application, researchers have continuously been investigating various techniques to improve performance, which may include enhancing the transducer’s sensitivity for sensor and/or receiver applications or amplifying the acoustic field generated for actuation and/or transmission [2]. To achieve this, the effects of changing the shape and geometry of the transducer as well as its configurations have been studied [7,8,9,12,13,15,18,19,20,21,22,23,24]. As an example, a curved plate improves the focus of the radiated waves but results in in-plane and flexural vibration mode coupling, which restricts the design and application [13,15,22]. Avoiding this obstacle and mode coupling necessitates changing other physical parameters and dimensions of the transducer, which could be cumbersome and impossible in certain circumstances. Another example can be a circular array configuration, which also has limitations, as any slight change in design or dimensions will affect the focal line and the transducer performance [9]. 

An alternative to the above approaches is a cylindrical shell, as it can improve the focus of the radiated waves and amplify the acoustic field. Further, for applications where a fluid medium is involved, a cylindrical geometry allows for the transducer to be installed in line with the incoming flow for liquid or gas sensing/actuation applications as the fluid passes through the transducer. These are some of the advantages of cylindrical geometry, making it a suitable alternative for many applications. Cylindrical shells of various materials have been used in the literature for different purposes. For instance, a cylindrical aluminum shell was excited by a piezoelectric shaker for drying fruits through the acoustically enhanced evaporation of the fruit’s water content [8]; however, it required an expensive and bulky power amplification system with a relatively high power supply (75 W). This necessitates some sort of modification to the transducer to cut down on the power requirements while increasing the acoustic output. 

For flat plate geometries, it was shown that a stepped-thickness configuration can lead to in-phase radiation from various regions of the plate that would have otherwise been out-of-phase [12,18,19,20,25,26]. Further, a combination of the curved focusing geometry of a cylinder and a stepped-thickness configuration to further eliminate the counter-phase radiation from various regions of a transducer seemed to have the potential for transducer applications where a focused amplified acoustic field with a relatively low power supply is desired. To this aim, both axially and circumferentially stepped-thickness cylindrical shells were investigated, proving that a suitably designed configuration of these steps can localize deformation within thin sections, control the mode of vibration and amplify the generated acoustic field at a low power input [2,4,5,27,28,29,30,31,32,33]. Further research revealed that these transducers can be effectively employed for applications in biomedical areas; an example is for airway humidification or drug delivery purposes [3]. 

Based on the above literature and considering the advantages outlined, it is evident that cylindrical transducers have a strong potential for numerous sensing and actuation applications. However, to the best of the authors’ knowledge, there are only a few studies available, and the literature lacks investigations and published data on the design and applications of these transducers as sensors and/or actuators. Hence, their potential seems not to have been fully investigated and employed. Further, since ceramic-based PZTs have been one of the most frequently and widely used piezoelectric materials among the four groups of piezoceramics, piezocrystals, piezopolymers and piezocomposites [3,4,5,6,7,8,9,10,11,12,13,14,15,16,17,18,19,20,21,22,23,24,25,26,27,28,29,30,31,32,33,34,35,36], the present work focuses on PZT cylindrical transducers. However, the readers may refer to many other available literature on piezopolymers, piezocomposites or other types of piezoelectric materials employed as actuators or sensors, such as [35,37]. Hence, the aim of this paper is to shed light on the potential of cylindrical PZT transducers, introduce their available applications, including at the research and lab scales, and finally propose some venues for future investigations.

## 2. Cylindrical Transducers

This section introduces various cylindrical transducers investigated and employed for different applications. Design considerations, modifications and operating performance will be elaborated on. Some of the applications of uniform-thickness cylindrical transducers will be presented, followed by modifications introduced in the transducers, such as stepped-thickness variations, tuning notches, etc. 

### 2.1. Uniform-Thickness Transducers

Cylindrical transducers can be used as an actuator for ultrasonic particle separation for various purposes. The hollow geometry of a cylinder makes it an attractive choice for particle separation in a dynamic flow. Additionally, the curved cylinder surface subject to a suitable vibration mode shape can result in a focused, stronger acoustic field inside the transducer. However, limited literature has investigated the use of cylindrical ultrasound transducers for particle separation. A cylindrical PZT-4 transducer with an outer diameter of 19 mm, inner diameter of 16 mm and length of 28 mm was employed for separating microparticles in a continuous flow system [38]. A water-based and a blood-resembling fluid were considered in the study. At 202 kHz, the transducer generated a flow instability, mixing the suspension instead of separating it. However, at 345 kHz, separation was achieved successfully. This verifies that the implementation of cylindrical transducers for acoustic separation requires identifying a suitable frequency and acoustic pressure level commensurate with the medium and particle properties. The separation of particles is achieved through the acoustic standing wave applied by the transducer to the fluid that is created from the interaction of the incident and reflected waves in a medium [39]. The standing wave has a region of maximum displacement (pressure nodes) and a region of minimal displacement (pressure antinodes). The particles are aligned depending on their acoustic contrast factor, which is a function of the particles’ density, medium (fluid) density, particle compressibility and fluid compressibility [40]. When the particles have a positive acoustic contrast factor, they move towards a pressure node and a negative acoustic contrast factor causes them to move towards pressure antinodes [41]. The extraction of microparticles can be performed through a customized collector in-line with the nodal and/or anti-nodal circles where particles gather depending on the acoustic contrast factor. 

**Figure 1:**
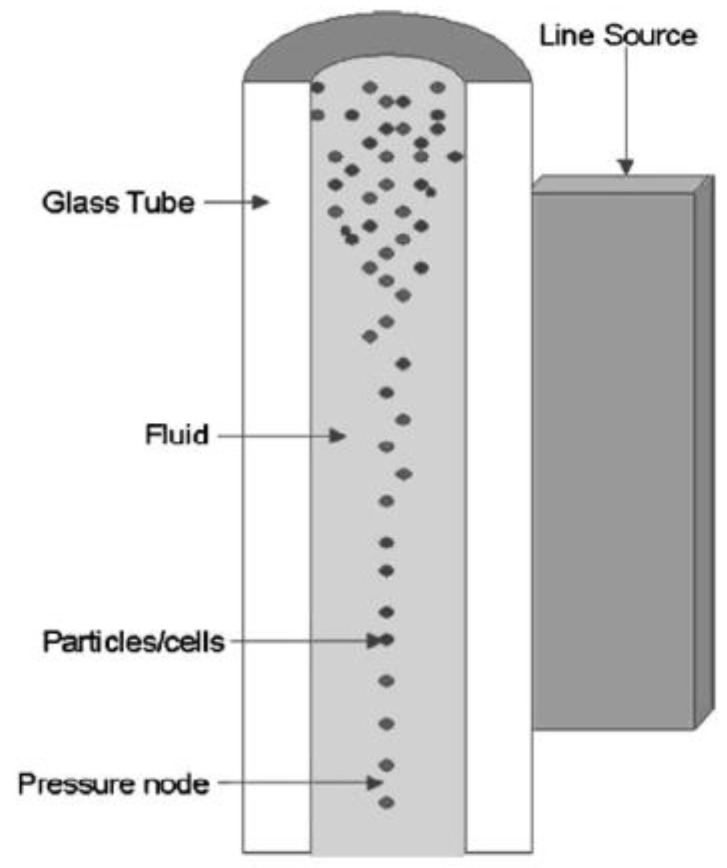
Glass cylindrical shell driven by a line-source piezoelectric transducer for particle concentration in a fluid medium [42].

Goddard and Kaduchak investigated the acoustic concentration of particles in a fluid-filled, long cylindrical glass tube driven by a piezoceramic with a line contact, as in Figure 1 [42]. The material properties and aspect ratio for the cylinder were chosen so that the breathing mode resonance frequency of the cylinder matched the resonance frequency of the fluid-filled cavity with one pressure node along the central axis for the concentration of particles, as illustrated in Figure 1.

The PZT4A piezoelectric transducer, with 30 mm length, 3 mm thickness, 1.5 mm width and a thickness mode resonance, was driven at 417 kHz, 10–12 V_pp_ and 80 mA at approximately 0.8–0.9 W. The polystyrene particles were 10 µm in diameter when diluted in water and were successfully concentrated within approximately 5 s along the central axis of the cylinder with an inner diameter of 2.2 mm, outer diameter of 3.97 mm and 15 cm of length.

In another application for fruit drying, an aluminum cylindrical shell (with an internal diameter of 100 mm, height of 310 mm and thickness of 10 mm) was excited at 21.8 kHz by a piezoelectric shaker at a suitable mode shape, generating a focused and stronger acoustic field at 155 dB using 75 W of electrical power, which is relatively high [8]. Although it was proven successful in improving the drying rate, a bulky and expensive power supply was required [43]. Reducing the power requirements and further amplifying the acoustic field are necessary for enhancing the uptake of such cylindrical transducers for various applications. One approach to achieving this is by making modifications to the shape and geometry of the transducer, as investigated in some studies using different methods, which will be elaborated on in the subsequent sections.

### 2.2. Modified Transducer Configurations

This section covers approaches available in the literature for modifying the cylindrical transducer configuration to improve performance. For instance, for the levitation of water droplets in the air, cylindrical transducers can be employed. The selection of the vibration frequency of the transducers strongly affects the generation of the acoustic standing wave inside the transducer with pressure nodes. To match the resonance frequency of the hollow cylindrical transducer with that of the cavity inside, two methods were proposed and investigated: (1) a physical change in the transducer geometry, and (2) a change in the resonance frequency of the cavity within the transducer [44,45,46]. These are elaborated on in the following sections.

#### 2.2.1. Structurally Tuned Transducer Configuration

**Figure 2:**
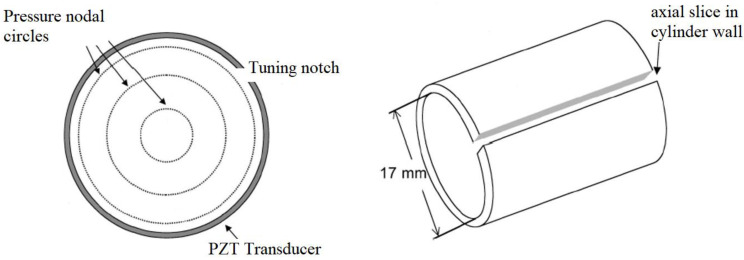
Structural tuning of the piezoelectric transducer by cutting a notch along the length (pressure node circles are illustrated as well); two views are depicted [45].

The first approach involves structural tuning of the cylinder by creating a cut along its length, as shown in Figure 2. This causes the breathing resonance frequency of the transducer to match one of the resonance frequencies of the cavity with a certain number of nodal circles inside the transducer, as depicted in the figure with three nodal circles.

**Figure 3:**
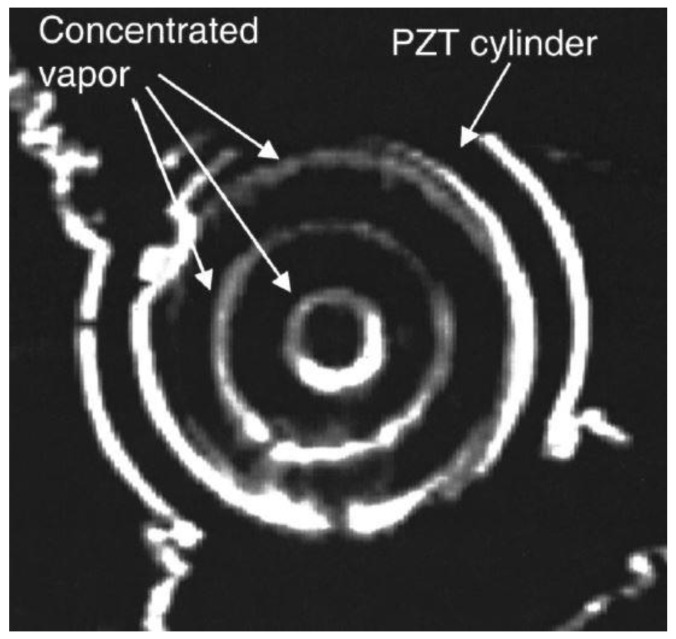
Effective concentration of water vapor at the nodal circles inside the PZT cylindrical transducer with a cut along the length [45].

The PZT transducer was radially poled with nickel electrodes on the outer and inner surfaces. The inner diameter was 16.9 mm, the outer diameter was 19.0 mm and the length was 17.0 mm, with the transducer being driven at 66.7 kHz, close to the third cavity mode of 65.7 kHz. The drive voltage was approximately 1 V_pp_ at approximately 100 mW of input power. The initial breathing mode frequency of the transducer was 61 kHz, which was altered to 66.7 kHz by adding the axial cut to match that of the cavity at its third mode of 65.7 kHz. The results revealed that the concentration of water droplets at the nodal circles can be achieved, as shown in Figure 3. 

#### 2.2.2. Cavity-Tuned Transducer Configuration

**Figure 4:**
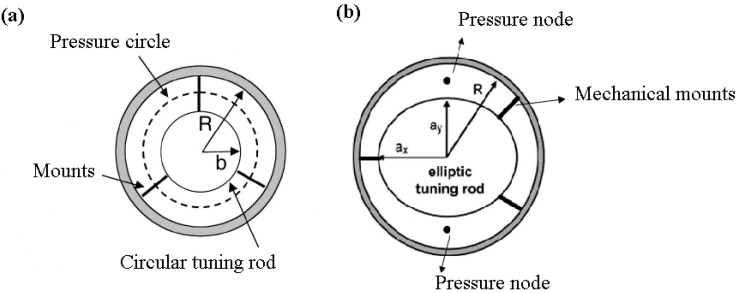
Cavity tuning in a piezoelectric transducer by inserting a circular (**a**) and elliptical (**b**) tuning rod inside the transducer [44,45].

The second approach, cavity tuning, involves altering the resonance frequency of the cavity inside the transducer to match the breathing resonance frequency of the transducer. To do this, various approaches were investigated, such as coaxially inserting an elliptical or circular metallic rod inside the transducer cavity, as shown in Figure 4 [44,45,46]. The rod diameter in the case of a circular insert is chosen to have one coaxial circular pressure node in the cavity, as illustrated in Figure 4a. For a radially poled cylindrical PZT of inner radius 1.96 cm, length 5.06 cm and outer radius 2.22 cm, operating at a breathing mode resonance of 23.2 kHz, the resonance in air occurs at R = 1.224 cm. The difference between this radius and the inner radius is approximately 0.73 cm, which is close to half the wavelength in the air at that frequency, leading to the formation of one circular node, as seen in Figure 4a.

The second case involved employing an elliptical cross-section with major and minor diameters of 25.3 mm and 24 mm, respectively, to break the axial symmetry of the cavity, which results in the formation of two localized pressure nodes (rather than circles) in line with the minor axis of the ellipse, as illustrated in Figure 4b. 

For collection purposes, a tube was placed at the end of the cavity around and in line with the localized pressure nodes and/or circles to collect water droplets. 

### 2.3. Stepped-Thickness Transducer Configurations

**Figure 5:**
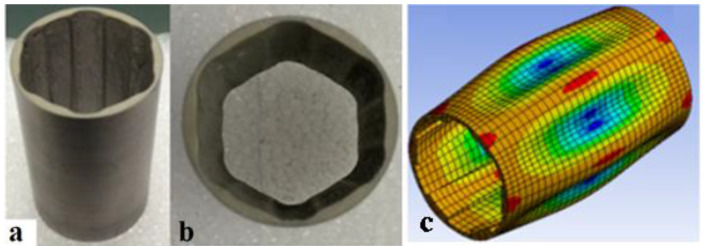
Circumferentially stepped-thickness piezoelectric transducer in two views (**a**,**b**); vibration mode shape with circumferential and axial mode numbers of 6 and 1, respectively (**c**) [2].

As stated earlier, to employ the benefits of the curved and focused geometry of a cylindrical transducer as well as stepped-thickness characteristics for flat plates, which had shown advantages over uniform-thickness plates, previous work investigated both circumferentially and axially stepped-thickness piezoelectric PZT cylindrical transducers in an effort to amplify the generated acoustic field inside the transducer without the need to increase the input power [2,4,5]. This required a suitable constructive interference of the radiated waves inside the transducer and minimized the counter-phase vibrations at any cross-section along the length. This required a vibration mode shape with suitable circumferential and axial mode numbers to eliminate or diminish out-of-phase vibrations and radiations. To achieve this, steps were introduced at equal intervals around the circumference to excite certain vibration mode shapes and create localized high vibration amplitudes. Figure 5 depicts an embodiment of a radially poled circumferentially stepped-thickness cylindrical PZT-5 transducer (with an outer diameter of 30 mm, an inner diameter of 26 mm and a length of 50 mm, driven at 31 kHz and having circumferential and axial mode numbers of six and one, respectively) in two views having six circumferential steps [2]. The delivered output acoustic pressure was, on average, more than 1.5 times greater than that of the identical uniform-thickness transducer driven at the same input power of 4.5 W.

It is also worth mentioning that these thickness variations can lead to stress concentration, which needs to be considered in the design of these transducers to eliminate design problems while having strong acoustic fields. The authors employed FEM analysis to investigate the stress distribution, vibration and acoustic response of these transducers. Both externally and internally stepped transducers with odd and even numbers of steps were investigated. Those with an even number of steps showed better constructive interference of the radiated waves and a more symmetrical acoustic field inside the transducer. It was also concluded that the uniformity of the acoustic field along the length of the transducer was favorable, with slight variations in the sound pressure level. This was attributed to the suitable vibration mode shape identified with an axial wave number of one leading to the whole length to vibrate in-phase longitudinally, as shown in Figure 5c. Another observation was that the frequency of operation at the intended mode shape was lower than that of the uniform-thickness or axially stepped one. This is also favorable for the long-term performance of the transducers. In fact, as a general rule, higher frequencies tend to cause overheating or gradual depolarization of the material [47] and possibly fatigue failure [20] (particularly in sharp edges, such as in stepped-thickness structures). Thus, a lower frequency is desirable to minimize overheating and depolarization. 

**Figure 6:**
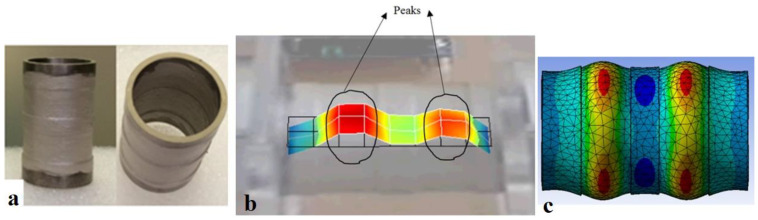
(**a**) Axially stepped-thickness transducer in two views; vibration mode shape with circumferential and axial wave numbers of zero and five, respectively: (**b**) Experimental; (**c**) Simulation [4].

Axially stepped cylindrical transducers were also investigated in the literature [4,5]. To identify the step configuration and frequency range, the authors first investigated the mode shapes and corresponding frequencies that have regions in counter-phase axially, i.e., modes with axial wave numbers of three and five. The stepped regions were introduced at the counter-phase region between each two consecutive in-phase regions. As an example, for the mode with an axial wave number of three, there was one region in counter-phase with the other two regions. Hence, one stepped-thickness region was introduced. Accordingly, this clarified the width of the stepped region. Similar to the circumferential stepped transducers, the aim was to have mode shapes without or with minimal out-of-phase vibrations and to localize higher amplitude vibrations to achieve an amplified acoustic field. Following these, the authors performed the analysis and achieved the embodiment depicted in Figure 6 with two axial steps for a radially poled PZT-5 transducer of an outer diameter of 30 mm, an inner diameter of 26 mm and a length of 50 mm driven at approximately 43 kHz and 4.5 W of input power. The output sound pressure obtained was nearly twice that of the identical uniform-thickness cylindrical transducer driven at the same input power.

**Figure 7:**
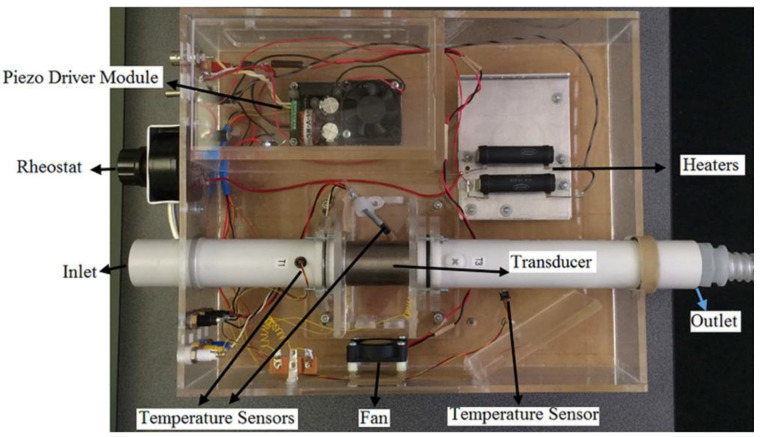
The experimental set-up includes a stepped-thickness cylindrical transducer to acoustically enhance evaporation of a stream of polydisperse microwater droplets [3].

Both the axially and circumferentially stepped transducers previously investigated [2,4,5] were designed for biomedical applications and humidification in lung therapies where it is preferable to have an in-line humidifier fitting in a breathing tube of approximately 2 cm diameter, such as those used in continuous positive airway pressure (CPAP) devices [1,48]. Accordingly, the authors investigated the effect of the focused acoustic field from these stepped-thickness transducers on a stream of polydisperse microwater droplets generated by a nebulizer [3]. Figure 7 illustrates the experimental set-up used in their study. Another requirement for this application based on the literature was that the sound pressure level (SPL) should exceed a threshold of 160 dB to be influential on the droplet size [43]. All these requirements were considered for identifying and designing a suitable stepped-thickness cylindrical PZT transducer [2,4,5]. 

The underlying mechanism exposed the droplets to the acoustic field while they passed through the transducer. This improved the relative movement between water droplets and air, leading to enhanced heat transfer and evaporation. Larger droplets in the stream were found to be affected more than small ones by the acoustic field. A noticeable reduction in the size of 90% of the droplets was observed, which improved the droplet size distribution in the stream. This was reported to be of particular and practical importance for some drug delivery applications or humidification in lung-supportive devices.

## 3. Discussion

As per the above sections and details, cylindrical transducers may have various applications in different areas, including but not limited to biomedical applications, the food industry, etc. However, their full potential has not yet been proven because of the limited number of available literature on cylindrical transducers as sensors or actuators. Nonetheless, the limited literature shows that researchers have managed to implement cylindrical transducers with some modifications to their design to suit their intended applications. Examples of such modifications were elaborated on along with their specifications, design considerations and operation principles. The hollow geometry of a cylindrical transducer allows for dynamic flow applications and stronger and more focused acoustic fields inside the transducer. Considering these benefits and advantages of a cylindrical geometry, researchers initially employed cylindrical structures composed of glass or metallic ones and drove them using some equipment and power drivers, which were bulky, expensive and power demanding as stated before [8,43]. Therefore, efforts were made to reduce power requirements by employing piezoelectric cylindrical transducers, which could be driven using a piezodriver, such as that used by [2,3,4,5], which had a very low input power and a compact, inexpensive driver unit. In terms of performance and design, various methods exist, some of which were introduced, including cavity tuning or structural tuning for synchronizing the resonance of the transducer with the cavity inside to enhance particle separation and concentration, as in [44,45,46]. The structural tuning discussed in these studies involved making a cut along the length, which might not be a suitable option for many applications as it leaves an opening along the whole length of the transducer, as shown in Figure 2. The other alternative, which was cavity tuning, involved the insertion of tuning rods of circular or elliptical cross-section inside the transducer using mechanical mounts. While this might work for some applications, it has its own barriers and disadvantages, as the insertions block part of the transducer’s cross-section, may reduce the flowrate, and may interfere with the incoming flow in some applications, leading to disturbance or turbulence. Hence, another alternative that does not have the above-mentioned restrictions and pitfalls is required. This was achieved by introducing circumferential or axial steps in the thickness of cylindrical piezoelectric PZT transducers [2,4,5]. The design has no limitation in terms of leaving an opening in the transducer geometry or blockage of the cross-section; hence, a strong potential for both sensing and actuation applications is provided in dynamic flow applications, for instance, as well as many other applications where the transducer can be installed externally and/or internally to the structure or geometry being triggered, excited or monitored. In addition to their low power requirements, they have proven to be capable of amplifying the generated acoustic field inside the transducer by approximately two times at the same input power of an identical uniform-thickness cylindrical transducer [2,4]. This might be of particular importance for many actuation applications. The design technology was employed for the dynamic flow actuation of a polydisperse stream of microwater droplets, as discussed earlier and resulted in improvements in the evaporation of droplets and enhanced droplet size distribution, which may be of particular interest for drug delivery or airway humidification purposes [3,27]. The strong potential of these stepped-thickness cylindrical piezoelectric PZT transducers has recently been explored and is currently being investigated and developed by the authors for some other sensing and actuation applications (the results will be published in due course), including but not limited to acoustic particle separation in the food industry (actuation) and picking up vibrations for structural monitoring (sensing). While these may not be the only alternatives to modify cylindrical transducers and tune them for various sensing and actuation applications, they are some examples of how it might be possible to devise them and make design modifications to generate strong acoustic fields and/or a certain number of pressure nodes or circles inside the transducer to be able to achieve the required performance for actuation or to alter the sensitivity of the transducer for sensing applications. 

## 4. Concluding Remarks

The aim of this paper was to shed light on the potential of cylindrical PZT transducers for various applications, such as actuators or sensors. Though not that plentiful, investigations available in the literature on cylindrical transducers—stepped thickness, structurally tuned and cavity-tuned transducers—were elaborated on in detail, discussing design considerations, requirements, applications and their performance. All these have shown a strong potential, however, there are some disadvantages and obstacles for structurally and cavity-tuned transducers in certain applications that were discussed, hindering their versatility. Further, the advantages, potential and available applications of axially and circumferentially stepped-thickness configurations were outlined, making them a suitable candidate for the future research trend on devising cylindrical PZT transducers that meet the requirements for a variety of applications in the biomedical, food and other industrial sectors. Alternatively, any other method capable of amplifying the acoustic field for actuation or improving sensitivity for sensor/receiver applications might be suitable for future research trends commensurate with the requirements and restrictions. 

## Data Availability

Not applicable.
